# Unveiling Insights: Harnessing the Power of the Most-Frequent-Value Method for Sensor Data Analysis

**DOI:** 10.3390/s23218856

**Published:** 2023-10-31

**Authors:** Victor V. Golovko, Oleg Kamaev, Jiansheng Sun

**Affiliations:** Canadian Nuclear Laboratories, 286 Plant Road, Chalk River, ON K0J 1J0, Canada; oleg.kamaev@cnl.ca (O.K.); jiansheng.sun@cnl.ca (J.S.)

**Keywords:** sensor data analysis, most-frequent-value, thermoluminescent dosimeters, environmental gamma background, dark matter, bootstrapping, confidence intervals

## Abstract

The paper explores the application of Steiner’s most-frequent-value (MFV) statistical method in sensor data analysis. The MFV is introduced as a powerful tool to identify the most-common value in a dataset, even when data points are scattered, unlike traditional mode calculations. Furthermore, the paper underscores the MFV method’s versatility in estimating environmental gamma background blue (the natural level of gamma radiation present in the environment, typically originating from natural sources such as rocks, soil, and cosmic rays), making it useful in scenarios where traditional statistical methods are challenging. It presents the MFV approach as a reliable technique for characterizing ambient radiation levels around large-scale experiments, such as the DEAP-3600 dark matter detector. Using the MFV alongside passive sensors such as thermoluminescent detectors and employing a bootstrapping approach, this study showcases its effectiveness in evaluating background radiation and its aptness for estimating confidence intervals. In summary, this paper underscores the importance of the MFV and bootstrapping as valuable statistical tools in various scientific fields that involve the analysis of sensor data. These tools help in estimating the most-common values and make data analysis easier, especially in complex situations, where we need to be reasonably confident about our estimated ranges. Our calculations based on MFV statistics and bootstrapping indicate that the ambient radiation level in Cube Hall at SNOLAB is 35.19 μGy for 1342 h of exposure, with an uncertainty range of +3.41 to −3.59μGy, corresponding to a 68.27% confidence level. In the vicinity of the DEAP-3600 water shielding, the ambient radiation level is approximately 34.80 μGy, with an uncertainty range of +3.58 to −3.48μGy, also at a 68.27% confidence level. These findings offer crucial guidance for experimental design at SNOLAB, especially in the context of dark matter research.

## 1. Introduction

SNOLAB [1,2], located beneath the Creighton nickel mine in Ontario, Canada, is a critical hub for conducting ultra-sensitive physics experiments that demand minimal background radiation. Its unique features, including a complex layout and depth of 2 km underground, make it an ideal environment for cutting-edge research. The facility, originally established for the Sudbury Neutrino Observatory (SNO) [3], now hosts numerous experiments. Notably, DEAP-3600 [4,5,6,7,8], the world’s largest liquid argon dark matter detector, operates in Cube Hall, alongside the MiniCLEAN [9] (now decommissioned) and the NEWS-G [10] (now at the commissioning stage) experiments. These endeavors primarily focus on detecting interactions between weakly interacting massive particles (WIMPs) and argon nuclei, offering insights into dark matter, which comprises a significant portion of the universe’s mass. Overall, SNOLAB’s contributions are crucial to advancing the understanding of fundamental physics and unraveling the mysteries of the cosmos.

The difficulties of searching for rare events, such as dark matter, include reducing unwanted background noise and improving the ratio of useful signal to unwanted noise in order to detect these uncommon events. This involves dealing with statistical limitations, the sensitivity of the equipment, and being able to differentiate between actual signals and noise. One specific type of noise is the gamma radiation from the surrounding environment that affects the detector.

Various methods are employed to assess low-level gamma-ray background in underground laboratories. For instance, at SNOLAB’s Cube Hall, external gamma radiation characterization techniques for the DEAP-3600 detector are detailed [11]. DEAP-3600 is a liquid argon dark matter detector with tight control over radioactive backgrounds [12]. It is placed in a water tank to shield it from rock radioactivity and cosmic ray muons.

Thermoluminescent detector (TLD) systems, functioning as passive sensors, are valuable for background monitoring in places such as Cube Hall because they are cost-effective, compact, and non-intrusive. They help detect any background variations that might affect measurements. Measurements around DEAP-3600’s water shielding have been performed using TLDs [11].

The uniformity or non-uniformity of environmental gamma-ray backgrounds in underground labs can result from various factors. This study employed TLDs similar to those used at Chalk River Laboratories (CRL) to measure doses. The assessments of the doses and dose rates with TLDs undergo regular independent blind tests [13,14] and have been successful.

The water shielding for DEAP-3600 is designed based on extensive Monte Carlo studies to optimize background rejection and environmental gamma background protection. These studies assume a uniform background distribution. This paper reports on external dose measurements in SNOLAB’s Cube Hall using TLD dosimeters placed around DEAP-3600’s water shielding.

### Scope of Work

In prior research [11], the effectiveness of employing integrating passive detectors such as TLDs to measure ambient radiation levels in SNOLAB, an underground facility with extremely low background radiation, was established successfully. Moreover, the research found that the background radiation does not spread uniformly around the water shielding of the DEAP-3600 detector. This discovery holds importance for both Monte Carlo simulation studies and the design of shields for upcoming large-scale dark matter detectors [15,16,17].

In this study, our primary objective was to confirm the variations we previously observed in the data with a high degree of confidence. We wanted to establish reliable confidence intervals for the non-uniform background radiation based on the data we have collected. Therefore, in the next section, we present our findings and the methodology of the data analysis, which underscores the significance of using Steiner’s most-frequent-value method in combination with bootstrapping.

## 2. Materials and Methods

### 2.1. Integrating Passive Detectors

This section offers a concise explanation of how passive detectors, in particular TLDs, are used as sensors to measure the overall environmental exposure to direct gamma radiation. More detailed information about TLDs and the specific method for calculating ambient doses from them are covered in another paper [11]. In this study, we used TLD-100s to measure environmental doses from photons. These detectors are mounted on dosimeter plaques and inserted into badges. The badges include aluminum filters to measure gamma radiation accurately and prevent dust and light interference.

Control dosimeters, kept alongside the DEAP-3600 detector in a well-shielded location, monitor doses unrelated to the test exposure, such as background radiation and transport or storage doses. These control dosimeters are subtracted from the deployed dosimeters’ readings to determine the net ambient dose accurately.

Quality assurance and control programs, in compliance with Canadian regulatory requirements [18,19], are implemented to ensure the precision and reliability of dose estimates. Annual independent blind tests [13,14] validate the accuracy of the results from CRL Dosimetry Services.

Despite the expectations of a low gamma background in Cube Hall at SNOLAB, rigorous measures are taken to improve measurement quality, including using two TLD-100 chips in the same badge casing for cross-verification. If ambient dose values deviate significantly, they are excluded from the analysis. Additional specific information about the criteria for excluding dose values from the analysis can be found in a separate publication [11].

### 2.2. Most-Frequent-Value Approach

Usually, the mode in statistics [20] is the value that appears most frequently in a dataset, often referred to as the dataset’s “peak”. It is like finding the most-common number. Some datasets might have more than one mode if multiple values have the same highest frequency. To determine the mode, one arranges the data and counts how often each value appears. The one with the highest count is the mode. If several values share this highest count, a multimodal dataset exists. If all values appear equally often, there is no mode. The mode is just one of the measures of central tendency used to describe a dataset’s typical or central value. The mode, alongside the mean and median, helps describe the dataset’s typical value and central tendency [20].

An alternative approach could involve using the most-frequent-value (MFV) method [21,22,23], as demonstrated in recent studies. The MFV approach, based on minimizing information loss, has been successfully applied in various fields [24]. Both the traditional mode and the MFV statistic are used to find the most-common value in a dataset, but they have a key difference. Consider this dataset: 5, 7, 9, 7, 8, 7, 4, 15. The mode is 7 since it appears three times, more than any other number. Now, alter the data slightly: 5, 6.9, 9, 7, 8, 7.1, 4, 15. Here, the old mode, 7, no longer works because no number appears more frequently than the rest. Although the values still center on 7, they are more spread out. The example provided here is a basic made-up dataset used to demonstrate the distinction between the mode and MFV in statistics. To explore more-recent and intriguing datasets, as well as the benefits of using the MFV approach compared to other statistical methods, one can refer to the following sources: Zhang (2017) [24], Zhang (2018) [25], Zhang et al. (2022) [26], and Golovko (2023) [27].

Steiner’s MFV method [21,22,23] can handle this situation. It identifies the most-common value, even if it is not the same as the traditional mode. In simple terms, the MFV helps find the most-common value, even when the data are somewhat scattered. This example illustrates the difference between the traditional “mode” and the “MFV” method in statistics.

Steiner’s MFV approach was used to analyze geophysical sounding logs in shallow, dry sediments [28]. This approach enhanced the analysis by employing an improved method that iteratively re-evaluated the data using factor analysis [29]. In addition, the MFV method has proven effective in groundwater modeling as a geostatistical technique [30]. Notably, it was used to determine the Hubble constant, irrespective of the data distribution [25], and addressed challenges in nuclear astrophysics, such as the lithium abundance problem [24]. The MFV method improves neutron lifetime estimation with non-Gaussian data [26]. Recently, a method, based on the MFV approach combined with bootstrap analysis, was used to provide a more-robust way to estimate historical measurements of ${}^{39}$Ar’s half-life [27]. This method has resulted in the uncertainty being a factor of three smaller than that of the most-precise re-calculated ${}^{39}$Ar half-life measurements corresponding to the 68% confidence level.

The MFV statistical technique offers several advantages [31,32]. It remains robust even when dealing with non-normally distributed data or outliers. Moreover, it provides a reliable estimate by calculating weighted averages efficiently. The MFV method also automatically calculates scale parameters during iterations [33], allowing for optimal weight coefficients to be determined for any given dataset.

The explicit equation for the iterations to find the MFV (*M*) is as follows:

(1) $$M_{j+1}=\frac{\sum_{i=1}^{N}x_{i}\cdot \frac{\varepsilon_{j}^{2}}{\varepsilon_{j}^{2}+{\left(x_{i}-M_{j}\right)}^{2}}}{\sum_{i=1}^{N}\frac{\varepsilon_{j}^{2}}{\varepsilon_{j}^{2}+{\left(x_{i}-M_{j}\right)}^{2}}}.$$

In this equation, $M_{j+1}$ represents the value obtained in the (j+1)-th step of the iteration process to determine the MFV. $x_{i}$ corresponds to the *i*-th element in the dataset, while *N* represents the total number of elements in the dataset. In addition, $\varepsilon_{j}$ refers to the dihesion (also known as Steiner’s scale factor [33]). For the initial value of the iteration, one can use the mean value of all the data in the sample, denoted as $M_{\left(0\right)}=\frac{1}{N}\sum_{i=1}^{N}x_{i}$.

The equation describing the iterations for the dihesion $\varepsilon_{j+1}$ is as follows:

(2) $$\varepsilon_{j+1}^{2}=3\cdot \frac{\sum_{i=1}^{N}\frac{{\left(x_{i}-M_{j}\right)}^{2}}{{\left(\varepsilon_{j}^{2}+{\left(x_{i}-M_{j}\right)}^{2}\right)}^{2}}}{\sum_{i=1}^{N}\frac{1}{{\left(\varepsilon_{j}^{2}+{\left(x_{i}-M_{j}\right)}^{2}\right)}^{2}}}.$$

In this equation, $\varepsilon_{j}$ stands for the value we calculated in the previous step. The “dihesion” is what determines how important each data point is when calculating the MFV. When it is big, every data point is equally significant. When it is small, only the ones close to the MFV truly count. One can find information on how the dihesion parameter is determined in the books [22,23]. For a practical example of how the dihesion is derived, one can refer to the sources [21,29]. The initial value for $\varepsilon_{\left(0\right)}$ can be selected as $\varepsilon_{\left(0\right)}=\frac{\sqrt{3}}{2}\cdot \left(x_{max}-x_{min}\right)$, where $x_{max}$ and $x_{min}$ denote the maximum and minimum values within the complete dataset used for the MFV (*M*) estimation. Both equations, labeled as Equations (1) and (2), which are used to calculate the values of $M_{j+1}$ and $\varepsilon_{j+1}^{2}$ from the datasets, need to be satisfied at the same time.

The iteration threshold value in Equations (1) and (2) can be set to a suitably small value, for instance $10^{-5}$. In essence, this threshold value signifies the limit that must be achieved through successive iterations concerning the next difference $\left|M_{j+1}-M_{j}\right|$ in Equation (1). When dealing with many data, the MFV method may take more time to compute compared to other statistical methods. This is because the MFV method requires multiple iterations, while other methods may be faster since they do not need multiple iterations. It is important to think about the balance between accuracy and computation time when deciding which method to use for analyzing large amounts of data. In this study, the MFV method had a maximum of 80 iterations, which is longer than the mean value method, for example. However, it still took a reasonable amount of time to estimate the MFV.

Steiner (1988) [21] demonstrated that the iteration process described by Equations (1) and (2) approximates a value that characterizes the concentration of data, and as a result, it is referred to as the most-frequent value (*M*) [23].

For a symmetrical distribution, as with normally distributed data, Csernyák and Steiner [23] provided a simple formula for calculating the variance ($\sigma_{M}$) when *M* is determined according to Equation (1):

(3) $$\sigma_{M}=\frac{\varepsilon }{\sqrt{n_{\mathrm{eff}}}}.$$

In this equation, ε represents the dihesion (ε here is the convergence value of the iterations as defined in Equation (2)), and $n_{\mathrm{eff}}$ is the effective number of data points. The effective number is computed as:

(4) $$n_{\mathrm{eff}}=\sum_{i=1}^{N}\frac{\varepsilon^{2}}{\varepsilon^{2}+{\left(x_{i}-M\right)}^{2}}.$$

This formula provides a means to calculate the variance when estimating *M* for symmetrical distributions, as with normally distributed data. For instance, in both of the previously mentioned datasets, the most-common value is M=7.0, while the average is $\bar{x}=7.8$. If we remove the last value from both datasets (for example, if it is considered an outlier), the most-frequent value remains M=7.0, while the mean becomes $\bar{x}=6.7$. This demonstrates that the MFV approach is more robust and stable.

As mentioned earlier, the MFV technique and confidence interval bootstrapping were used to analyze neutron lifetime measurements. We used the original neutron lifetime data from a study Zhang et al., (2022) [26] as a test to check if the MFV and bootstrapping algorithms give consistent results. Even though the bootstrap process is random, we found that it is better to use a confidence level specificto the second decimal place (for example, 1 sigma corresponds to 68.27%) in order to replicate the MFV confidence interval for neutron lifetime measurements. Therefore, we adopted the same approach in this study.

### 2.3. Bootstrapping Approach for Confidence Intervals

In this section, we aim to outline a general approach for bootstrapping confidence intervals. In the next section, we will delve into the specific method used for analyzing ambient dose results.

Bootstrapping [34,35] is a statistical method for estimating the confidence interval of a sample statistic, such as the mean, or median, or MFV, when one does not know the population’s underlying distribution. Instead of making assumptions about the population, bootstrapping relies on resampling from observed data.

The bootstrapping technique operates in the following manner. Initially, it commences with an original dataset and subsequently generates numerous new datasets through the random selection of data points from the original dataset. This process permits some data points to be selected multiple times, while others may not be chosen at all. Subsequently, for each of these freshly generated datasets, a target statistic, such as the mean or most-frequent value, is computed. Consequently, this yields a compilation of these computed statistics, constituting a distribution, which serves as an approximation of the sampling distribution of the designated statistic. Finally, this distribution is employed to determine the range of values encompassing the statistic of interest at a specific confidence level, typically set at 95.45%. This derived range is formally recognized as a confidence interval [36,37].

Bootstrapping offers a robust methodology, especially when one is confronted with limited data or intricate scenarios where traditional statistical approaches may not be applicable. It allows researchers to make informed estimations based on observed data, thereby facilitating a deeper comprehension of the plausible range for the true population statistic.

## 3. Results

Detectors were positioned within Cube Hall at SNOLAB (see Figure 1 in [11]), with a focus on the DEAP-3600 water shield area (see Figure 4 in [11]). Table 1 presents data for detectors that met a specific selection criterion (which is explained later). It includes both “rear” and “front” TLD chips (see Figure 3 in [11]), along with their average values and standard deviations. These detectors were deployed on 27 November 2018 and removed on 22 January 2019, totaling 1342 h of exposure. The deployment and removal took 2 h, and this time was considered when measuring the interval. A calibration method recommended by NIST [38] was used to ensure that the timer’s accuracy was within 0.02%. However, the timer’s accuracy was not considered in the ambient dose rate measurements, as it was much smaller than the standard deviation in the dose measurements.

Accurately measuring radiation exposure, especially in environments rich in X-rays and gamma-rays such as Cube Hall at SNOLAB, poses a significant challenge [11]. This is due to the need to obtain precise data on the ambient radiation levels within Cube Hall, where radiation levels can vary considerably across different locations. This involves choosing the right data to determine the true ambient radiation for passive TLD sensors in the same badge.

To do this, statistical analysis considers variability within and between groups of TLD badges. These variations are seen as random effects and are described by their associated components of variance. For TLD badges with unique IDs, only two TLD chips were placed in the same spot in Cube Hall. Through calibration, it was confirmed that the measured doses from these chips in the same badge were consistent within their variance, serving as a selection criterion for the ambient dose.

Table 1 displays the results for TLDs that met this criterion, meaning the ambient dose results for the rear and front TLD chips in the same badge were within one standard deviation of each other. Control dosimeters detected 83.6±9.7μGy (9.5 ± 1.1 mR) of radiation during transportation and storage (information about how the control dosimeters are used can be found in [11]). We also provide the dose value in milliroentgens (mR) to align with the dose value previously published [11]. The data presented in Table 1 of the reference paper by Golovko et al. [11] were used for the results shown in Table 1. The only change made here was the conversion of the dose measurements into gray (Gy) units (8.8 μGy/mR [39,40]).

The TLD detectors with IDs from 4 to 24 were on the DEAP-3600 water shield, IDs 25 and 26 were near a fire door (see Figure 2 in [11]); ID 28 was on the Cube Hall deck; ID 29 was on top of the DEAP-3600 water shield. The specific locations within Cube Hall are not detailed in this paper.

In principle, one expensive and time-consuming option to obtain a confidence interval would be to replicate the TLD measurement at the DEAP-3600 water shield several times. If one repeats the experiment several times, then one can keep track of each ambient dose value and end up with a larger set of dose data, which could be used to estimate the confidence interval. However, as mentioned earlier [11], repeating the ambient dose measurements with multiple passive TLD sensors several times is both expensive and time-consuming, although it requires much less time compared with the use of active detectors.

Suppose one possesses a dataset [11] reflecting authentic ambient dose measurements (refer to Table 1) and one requires a confidence interval determined through statistical methods, such as the MFV. Instead of replicating ambient dose measurements multiple times, one can consider a bootstrap approach [36,37]. An additional advantage of bootstrapping is its applicability in cases with or without a well-defined probability model for the data [35]. Thus, let us use the bootstrapping technique to gain deeper insights into which confidence interval more accurately represents the ambient dose measurements at Cube Hall. As previously noted, the MFV technique and confidence interval bootstrapping were applied to perform a reliable analysis of neutron lifetime measurements [26]. In this work, we aimed to use the same approach to establish a confidence interval for ambient dose measurements at Cube Hall.

The average radiation levels were measured in two different situations at SNOLAB’s Cube Hall. In one scenario, we placed TLD sensors both at the front and rear positions, and these sensors were spread out around the water shielding of the DEAP-3600 detector. In another scenario, we used passive integrating sensors, again at the front and rear positions, and these sensors were positioned in two places: around the water shielding of the DEAP-3600 detector and inside Cube Hall itself. More information with pictures about the positions of the TLDs badges mentioned in Table 1 can be found in [11].

**Figure 1:**
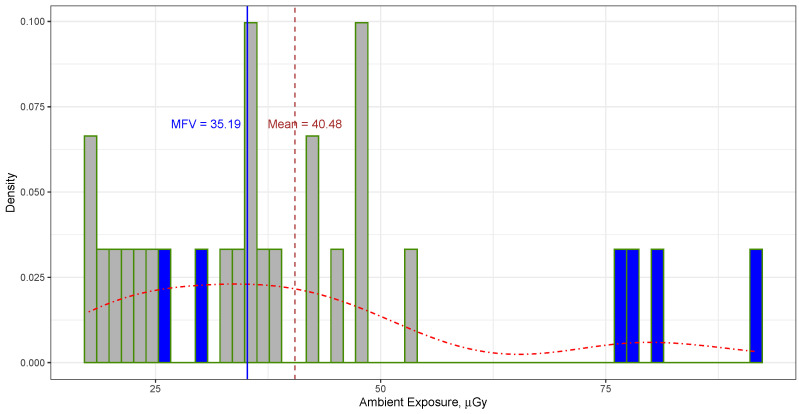
Histogram and probability density (represented by the dotted–dashed line) show the distribution of ambient dose data at Cube Hall (see Table 1). The vertical solid line marks the MFV, while the vertical dashed line represents the mean value. The ambient radiation levels outside of the DEAP-3600 water shielding are emphasized with a darker color.

The mean of all ambient dose measurements taken at the “front” and “rear” positions of the TLD sensors in Cube Hall at SNOLAB, as listed in Table 1, was calculated to be 40.48±20.03 μGy (or 4.6±2.3 mR [11]), with the error indicating the standard deviation. The quoted uncertainties are given at the 68.27% confidence level, which corresponds to the [20.45, 60.51] confidence interval, whereas the 95.45% confidence interval for all data is [0.42, 80.54]. The reason why the confidence level was chosen as 68.27% or 95.45% is because these values are widely used in statistical analysis and associated with 1 and 2 sigma errors. In contrast, the MFV for these measurements was determined to be 35.19±3.30μGy. It is important to note that this error reflects the variance of the MFV, as calculated using Equation (3). This equation assumes that the data follow a symmetrical distribution, which may not necessarily be true in this particular scenario. Therefore, it would be advantageous to employ a bootstrap approach to establish a reliable confidence interval with a high level of confidence. The mean and MFV values are shown as vertical lines in Figure 1.

**Figure 2:**
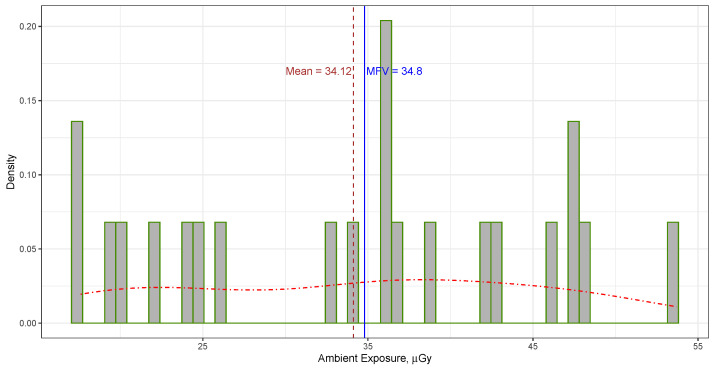
Histogram and probability density (represented by the dotted–dashed line) show the distribution of ambient dose data at the water shielding of the DEAP-3600 detector in Cube Hall (see Table 1). The vertical thin solid line marks the MFV, while the vertical thin dashed line represents the mean value.

The mean of the ambient dose measurements gathered from passive integrating sensors positioned at various locations of the water shielding surrounding the DEAP-3600 detector within Cube Hall at SNOLAB from both the front and rear TLDs, as indicated in Table 1, was computed to be 34.12±11.19 μGy (or 3.9±1.3 mR [11]). The provided uncertainties are expressed at the 68.27% confidence level, corresponding to the confidence interval [22.93, 45.31]. The 95.45% confidence interval for all the data is [11.74, 56.50]. In contrast, the MFV derived from these measurements was determined to be 34.80±3.36 μGy. It is crucial to emphasize that this error corresponds to the variance of the MFV, calculated using Equation (3). These values are visually depicted as vertical lines in Figure 2. It is worth noting that the MFV for the ambient dose around the water shielding of the DEAP-3600 detector is quite similar to the MFV ambient dose observed in Cube Hall.

In essence, the average statistic from the data in Table 1 can give us a confidence interval at both the 68.27% and 95.45% confidence levels. This information could help us estimate the maximum ambient radiation levels in Cube Hall and around the water shielding of the DEAP-3600 detector. However, there is a catch—the average statistic assumes that the data follow a normal (or Gaussian) distribution. As we discussed in Section 2.2, this method is not always reliable and stable.

Our research revealed a more-dependable approach: using the most-frequent value along with bootstrapping. This combination provides trustworthy estimates of the ambient radiation levels both at the water shielding surrounding the DEAP-3600 detector and within Cube Hall at SNOLAB. It offers an efficient alternative to traditional data-collection methods. Specifically, it helps us pinpoint the most-common values and simplifies the data analysis, which are crucial when we need precise ranges of values.

As we already mentioned, an alternative method is to use a statistical technique called bootstrapping. It is particularly useful when the original dataset lacks observational errors [41]. One repeats this bootstrapping process multiple times (typically 1000 to 3000 times) to create a distribution of a statistic called the MFV. From this MFV distribution, one can calculate confidence intervals at different confidence levels, such as 68.27% and 95.45%. It is important to note that gathering a comparable set of ambient dose data using the traditional method, as described in the previous study [11], would take an impractical 459 years (3000 tests, each taking 1342 h) using passive integrating sensors such as TLDs.

**Figure 3:**
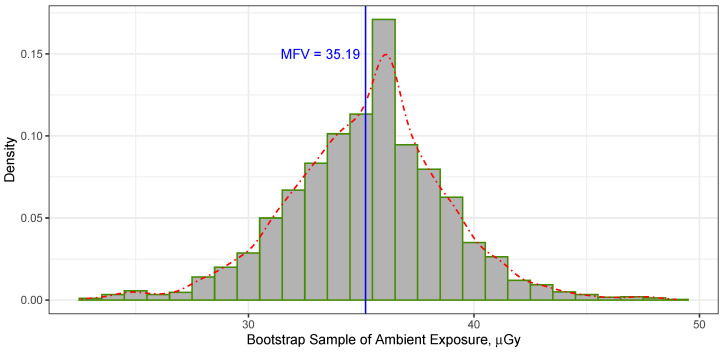
The histogram represents the MFV of the bootstrapped data (based on 3000 replicates), while the dotted–dashed line represents the probability density, illustrating the distribution of ambient dose data in Cube Hall. The vertical thin solid line indicates the MFV calculated from the data in Table 1.

Using the bootstrapping technique for all the ambient dose measurements taken at both the front and rear positions of TLD sensors in Cube Hall at SNOLAB, as listed in Table 1, along with the MFV statistics at the 68.27% confidence level, yielded a confidence interval for all the measurements of [31.60, 38.60]. Meanwhile, the 95.45% confidence interval for all the data was [28.10, 42.11]. The histogram in Figure 3 presents the MFV derived from 3000 replicates of the bootstrapped data. In simpler terms, estimating the ambient dose in Cube Hall at SNOLAB using the MFV statistics and a bootstrapping technique resulted in a value of

(5) $$D_{CH}\left(M\right)=35.19_{-3.59}^{+3.41}\;\;\mu \mathrm{Gy},$$

with a range of [31.60, 38.60], representing the uncertainty at the 68.27% confidence level. Comparing these bootstrapping errors to the MFV variance estimated using Equation (3), which resulted in $\sigma_{M}=3.30\;\mu$Gy, we found that the variance was of the same order as the bootstrap uncertainty indicated in Equation (5). The advantage of bootstrapping over the MFV variance is that the former does not rely on assumptions about the data distribution.

**Figure 4:**
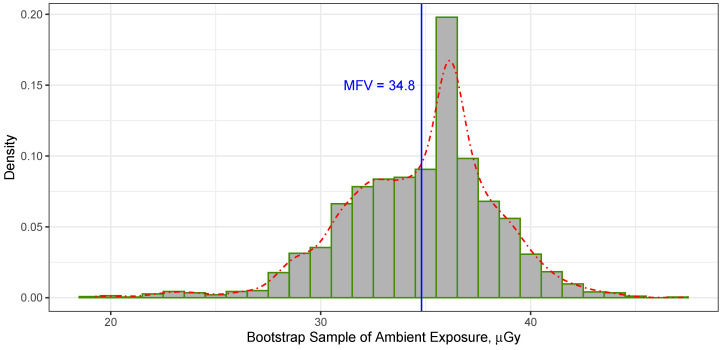
The histogram represents the MFV obtained from 3000 replicates of the bootstrapped data based on ambient dose data collected through passive integrating sensors positioned at different locations around the water shielding encircling the DEAP-3600 detector. In contrast, the dotted–dashed line illustrates the probability density. The vertical thin solid line serves as a reference point, marking the MFV calculated from the dataset listed in Table 1.

Applying the bootstrapping approach to the entirety of the ambient dose measurements gathered from the passive integrating sensors positioned at various locations of the water shielding surrounding the DEAP-3600 detector within Cube Hall at SNOLAB from both the front and rear TLDs, as indicated in Table 1, combined with the MFV statistics at a 68.27% confidence level, produced a confidence interval for these measurements within the range of [31.32, 38.38]. Furthermore, a 95.45% confidence interval for all the data was determined to be [27.79, 41.91]. The histogram featured in Figure 4 showcases the MFV derived from 3000 replicates of the bootstrapped data. In more-accessible terms, estimating the ambient dose of the water shielding surrounding the DEAP-3600 detector within Cube Hall at SNOLAB through the use of the MFV statistics in conjunction with the bootstrapping methods yielded a result represented as:

(6) $$D_{w.\mathrm{sh}.}\left(M\right)=34.80_{-3.48}^{+3.58}\;\;\mu \mathrm{Gy},$$

with an associated range of [31.32, 38.38], which denotes the level of uncertainty at the 68.27% confidence level. When comparing these bootstrapping errors to the MFV variance, as computed through Equation (3) and yielding $\sigma_{M}=3.36\;\mu$Gy, it becomes evident that the variance aligns with the magnitude of the bootstrap uncertainty presented in Equation (6). The advantage of using bootstrapping, as opposed to the MFV variance (see Equation (3)), is its ability to remain unaffected by assumptions about how the data are distributed, whether they follow a symmetric (balanced) or asymmetric (unbalanced) pattern.

In contrast, if one wants to determine the uncertainty of the MFV using Equation (3), there is no need to use the bootstrapping technique to measure the extent of the bootstrap uncertainty. However, one must possess prior knowledge that the observed measurements adhere to a Gaussian (or normal) distribution. This requirement is akin to what is needed for calculating the mean or weighted mean statistic. Nevertheless, it is important to note that data do not always follow a Gaussian distribution, even if the dataset is quite extensive [24,25,26,42]. For instance, studies have indicated that, if two independent variables adhere to a normal distribution, their ratio does not [43]. This clearly illustrates the limitation of applying standard normal statistics in certain physical scenarios.

Taking into account the 1342-h exposure of passive integrating TLD sensors and using the MFV ambient doses from Equations (5) and (6), we can compute the ambient dose rates within Cube Hall and around the DEAP-3600 water shielding at SNOLAB, yielding:

(7) $$R_{\mathrm{CH}}\left(M\right)=26.22_{-2.67}^{+2.54}\;\;\mathrm{nGy}/h\;\;\mathrm{and}\;\;R_{w.\mathrm{sh}.}\left(M\right)=25.93_{-2.59}^{+2.67}\;\;\mathrm{nGy}/h$$

with uncertainty representing the 68.27% confidence level. Specifically, the one-σ range for the ambient dose rate within Cube Hall, as deduced from MFV bootstrapping ($R_{\mathrm{CH}}\left(M\right)$), encompasses [23.55, 28.76], while the ambient dose rate encompassing the DEAP-3600 water shielding ($R_{w.\mathrm{sh}.}\left(M\right)$) lies within [23.34, 28.60]. Furthermore, the two-σ range for the ambient dose rate in Cube Hall, determined via MFV bootstrapping ($R_{\mathrm{CH}}\left(M\right)$), is [20.94, 31.38], and the corresponding range around the DEAP-3600 water shielding ($R_{w.\mathrm{sh}.}\left(M\right)$) is [20.71, 31.23]. Both of these ranges signify a 95.45% confidence level.

## 4. Discussion

In our prior research [11], we verified the effectiveness of passive detectors such as TLDs for measuring low-level radiation at SNOLAB. Moreover, we observed non-uniform environmental ambient doses around the water shielding. Table 1 emphasizes noticeable variations within one standard deviation of the outcomes, mainly influenced by the nearby MiniCLEAN water tank near the DEAP-3600 detector. These data are important for Monte Carlo simulations used to evaluate background levels for DEAP-3600 or any potential large-scale dark matter experiments.

Nonetheless, a lingering question was the determination of a highly accurate confidence interval for this non-uniform background radiation. Employing the most-frequent-value method in combination with bootstrapping, we tackled this inquiry and established suitable confidence intervals for both Cube Hall and the DEAP-3600 water shielding, achieving confidence levels of 68.27% and 95.45%.

The ambient dose and dose rate information can serve as cautious upper bounds for gamma radiation in the vicinity of the DEAP-3600 water shield. These data are instrumental in determining the required shielding thickness to ensure that detectors, such as DEAP-3600, remain unaffected by environmental gamma background. Specifically for DEAP-3600, the maximum ambient dose around the water shielding and the highest ambient dose rate are 41.91 μGy and 31.23 nGy/h, respectively, with a 95.45% level of confidence.

In a similar way, we can calculate the maximum expected levels of environmental gamma radiation in Cube Hall for any extensive detector setup. These calculations offer average values and statistical margins, essentially providing us with a safety buffer. For Cube Hall, in particular, the highest expected ambient dose is 42.11 μGy, and the maximum anticipated ambient dose rate is 31.38 nGy/h, both with a 95.45% confidence level. This method proves to be more efficient than deploying a single active detector at various spots one after the other, a process that would be significantly more time-consuming.

As we mentioned before, the unevenness of the radiation levels around the DEAP-3600 detector and in the Cube Hall at SNOLAB have been discussed in a previous study [11]. In this work, our main focus was to determine the range of uncertainty based on actual measurements using passive sensors like TLDs, with a specific level of confidence (for example, 68.27% or 95.45%). For future dark matter detectors, it would be helpful to know the upper limit of the uncertainty range, which can be used as a cautious estimate of the background radiation level for designing the shielding. Shielding against the background radiation can improve the accuracy of dark matter measurements by providing better control over the experimental conditions and reducing uncertainties. However, designing and implementing such shielding can be expensive and technically challenging. It may require advanced materials, sophisticated techniques, and additional infrastructure, which can increase the complexity and cost of the experiment. So, if we use passive sensors such as TLDs and specific statistical methods such as the MFV and bootstrapping, we can provide a range of uncertainty with a known level of confidence. This will give accurate and dependable results.

In summary, this study showcased the effectiveness of passive detectors such as TLDs as sensors in gauging minimal radiation levels at SNOLAB. It also furnished crucial information for evaluating the ambient radiation in the vicinity of the DEAP-3600 detector and within Cube Hall. In addition, the use of the MFV coupled with bootstrapping techniques enables the determination of confidence intervals with a high level of certainty. For direct dark matter detection experiments, such as DEAP-3600, which rely on extremely sensitive detectors, understanding the distinction between dark matter signals and background interference is paramount.

## 5. Conclusions

Using passive sensors such as TLDs and the MFV statistical method, we estimated the ambient radiation levels in Cube Hall and around the DEAP-3600 water shielding at SNOLAB. This approach provides a cost-effective and efficient alternative to traditional methods. The data are crucial for simulating how unwanted radiation might impact experiments, especially when searching for dark matter particles such as WIMPs.

Our calculations showed that, in Cube Hall at SNOLAB, the ambient radiation level, determined using the MFV statistics and bootstrapping, for 1342 h of exposure is approximately 35.19 μGy, with an uncertainty range of +3.41 to −3.59μGy, representing a 68.27% confidence level. Around the DEAP-3600 water shielding, the ambient radiation level is about 34.80 μGy, with an uncertainty range of +3.58 to −3.48μGy, also at a 68.27% confidence level. These estimates provide essential insights for experimental planning in SNOLAB, particularly for dark matter research.

We harnessed the bootstrapping technique to compute confidence intervals for our ambient dose measurements. This method is advantageous because it does not assume a specific data distribution and can be used with data that do not follow a normal distribution. By combining the MFV statistic with bootstrapping, we established confidence intervals at the 68.27% and 95.45% levels for ambient dose levels. This information is vital for evaluating the potential impact of environmental electromagnetic background radiation on large-scale dark matter experiments.

Moreover, this research showed that using the MFV method along with bootstrapping gives results similar to those of traditional methods, but in a much shorter time. We found that, gathering equivalent data using traditional approaches would be extremely time-consuming and impractical. These techniques are not just useful for measuring ambient dose and dose rates; they can also be used in other scientific fields where analyzing sensor data is necessary, especially when dealing with complex data distributions.

In conclusion, this study underscored the efficacy of employing the MFV and bootstrapping methodologies for precise ambient radiation level estimation, thereby furnishing invaluable guidance for radiation exposure control within facilities such as SNOLAB. These approaches present pragmatic and efficient alternatives to traditional data-acquisition methods. Notably, this research represents the first documented instance of using the most-frequent value in conjunction with bootstrapping techniques to calculate confidence levels with exceptional precision using TLDs as sensor detectors.

## Figures and Tables

**Table 1:** This dataset comprises measurements obtained from passive detectors, specifically TLDs, used for quantifying exceptionally low-level ambient radiation doses and dose rates at the surface of the water shielding of the DEAP-3600 detector. However, TLDs with Badge IDs 25 and 26 were an exception; they were positioned near the fire door. Furthermore, TLD detectors within Badge ID 28 were situated on the Cube Hall’s deck, while those in Badge 29 were positioned on top of the DEAP-3600 detector’s water shielding.

Badge ID	Rear Exposure (μGy)	Front Exposure (μGy)	Average Exposure (μGy)	Average Rate (nGy/h)
4	38.7	45.8	42.2 ± 5.0	31.5 ± 3.7
11	24.6	26.4	25.5 ± 1.2	19.0 ± 0.9
12	36.1	36.1	36.1 ± 0.0	26.9 ± 0.0
13	19.4	17.6	18.5 ± 1.2	13.8 ± 0.9
16	17.6	20.2	18.9 ± 1.9	14.1 ± 1.4
17	22.0	23.8	22.9 ± 1.2	17.0 ± 0.9
19	48.4	43.1	45.8 ± 3.7	34.1 ± 2.8
20	42.2	53.7	48.0 ± 8.1	35.7 ± 6.0
23	36.1	32.6	34.3 ± 2.5	25.6 ± 1.9
24	47.5	47.5	47.5 ± 0.0	35.4 ± 0.0
25	76.6	91.5	84.0 ± 10.6	62.6 ± 7.9
26	78.3	81.0	79.6 ± 1.9	59.3 ± 1.4
28	29.9	25.5	27.7 ± 3.1	20.7 ± 2.3
29	37.0	34.3	35.6 ± 1.9	26.6 ± 1.4

## Data Availability

Not applicable.

## Abbreviations

The following abbreviations are used in this manuscript:

CNL	Canadian Nuclear Laboratories
CRL	Chalk River Laboratories
DEAP	Dark matter Experiment using Argon Pulse-shape discrimination
ID	identification
MFV	most-frequent value
NEWS-G	New Experiments With Spheres-Gas
TLD	thermoluminescent dosimeter
SNO	Sudbury Neutrino Observatory
SNOLAB	a lab created to host other neutrino and dark
	matter experiments after the success of the SNO experiment
WIMP	weakly interacting massive particle
